# Immunoregulatory role of exosomal circRNAs in the tumor microenvironment

**DOI:** 10.3389/fonc.2025.1453786

**Published:** 2025-02-17

**Authors:** Cunming Lv, Jinhao Chen, Yuxiang Wang, Yichen Lin

**Affiliations:** ^1^ College of Basic Medical Sciences, China Three Gorges University, Yichang, China; ^2^ Hubei Key Laboratory of Tumor Microenvironment and Immunotherapy, China Three Gorges University, Yichang, China; ^3^ College of Electrical Engineering and New Energy, China Three Gorges University, Yichang, China; ^4^ Department of Medicine, Ningbo University, Ningbo, China

**Keywords:** exosomes, circular RNAs, tumor microenvironment, immunomodulation, tumor metastasis, signal pathway, tumor-associated macrophages

## Abstract

As cancer incidence and mortality rates rise, there is an urgent need to develop effective immunotherapy strategies. Circular RNA (circRNA), a newly identified type of non-coding RNA, is abundant within cells and can be released via exosomes, facilitating communication between cells. Studies have demonstrated that exosomal circRNAs can alter the tumor microenvironment and modulate immune responses by influencing the functions of T cells, natural killer (NK) cells, and macrophages, thereby enabling tumors to evade the immune system. Moreover, exosomal circRNAs show potential as diagnostic biomarkers and therapeutic targets for cancer. This review summarizes the regulatory roles of exosomal circRNAs in immune cells and their potential applications in cancer progression and treatment, highlighting their promise in improving cancer immunotherapy. Future research should concentrate on understanding the mechanisms of key exosomal circRNAs and developing targeted immunotherapy methods.

## Introduction

With the global rise in cancer incidence and mortality, there is an urgent need for more precise and effective treatments and the discovery of new therapeutic targets. Cancer immunotherapy has become a prominent research area, showing significant advancements and the potential to enhance treatment outcomes (1). This approach increases the antigenicity of tumor cells and boosts the cytotoxicity of immune cells, thereby enhancing the immune system’s capacity to eliminate cancer cells. Compared to traditional radiotherapy and chemotherapy, immunotherapy not only improves tumor response rates and extends treatment effectiveness but also significantly minimizes side effects. Consequently, cancer immunotherapy is increasingly seen as a promising option, especially for hard-to-treat cancers (2).

However, immune cells associated with tumors are pivotal in cancer development and progression. Their interactions with tumor cells can either promote or inhibit cancer, underscoring the complexity of their regulatory mechanisms (3). These mechanisms involve multiple signaling pathways governed by immune regulatory proteins that continuously operate within the tumor microenvironment (TME), significantly affecting tumor initiation, progression, and metastasis (4). Despite advancements, current cancer immunotherapies encounter major challenges, including the frequent development of drug resistance, which hinders effective treatment and decreases patient survival rates (5).

To address these challenges, recent research has focused on molecular entities within the TME that regulate immune responses. Non-coding RNAs, in particular, have attracted significant attention for their roles in gene expression regulation and cellular communication (6). Circular RNAs (circRNAs), a novel type of non-coding RNA, have become key regulators in various biological processes, including cancer progression and immune modulation. Unlike linear RNAs, circRNAs form covalently closed loops, granting them exceptional stability and resistance to exonucleases. This unique structure enables circRNAs to act as effective regulators by serving as microRNA (miRNA) sponges, interacting with RNA-binding proteins, and even influencing transcription and splicing (7).

At the same time, exosomes—tiny vesicles involved in intercellular communication—have been recognized as crucial carriers of circRNAs within the TME. These vesicles contain a wide variety of biomolecules, including DNA, mRNA, microRNA, circRNA, metabolites, lipids, cytoplasmic proteins, and surface proteins, enabling the transfer of these molecules between cells (8). The specific proteins on exosome surfaces can bind to receptors on target cells, delivering their internal contents through endocytosis or membrane fusion. Importantly, the incorporation of circRNAs into exosomes increases their stability and allows them to engage in long-distance communication between cells, thereby affecting the behavior of recipient cells within the TME (9).

Studies have shown that exosomal circRNAs can alter the tumor microenvironment and regulate immune responses related to tumors. For example, exosomal circRNAs can influence the activity and function of various immune cells, including T cells, natural killer (NK) cells, and macrophages, thereby aiding in immune evasion and tumor progression (10). Additionally, the abnormal regulation of circRNAs within exosomes has been linked to chemotherapy resistance, metastasis, and the suppression of anti-tumor immune responses. Due to their stable structure and specific expression patterns, circRNAs within exosomes are promising candidates for both diagnostic biomarkers and therapeutic targets in cancer (11).

This review seeks to examine the immune regulatory role of exosomal circRNAs within the tumor microenvironment, building on the essential foundation of cancer immunotherapy and the growing importance of circRNAs (12). We will discuss their immune regulatory potential from three angles: (1) Analyzing the sources and components of circRNAs in exosomes to understand their diversity and distribution within the tumor microenvironment; (2) Exploring the impact of circRNAs on tumor immune regulation, particularly their effects on immune cells and immunosuppressive signaling pathways; and (3) Evaluating the clinical application prospects of circRNAs, including their potential roles in tumor treatment and monitoring, as well as their feasibility for clinical translation (13).

This review systematically summarizes recent advancements in the role of exosomal circRNAs in tumor immune regulation, offering new insights and scientific evidence to support the development of more effective cancer treatment strategies in the future.

## Immune regulation in the TME

TME encompasses non-cancerous cells and components within a tumor, including various cell types, extracellular matrix (ECM), and biomolecules, creating a complex and diverse ecosystem (14). For example, the TME of pancreatic ductal adenocarcinoma (PDAC) includes multiple cell types such as pancreatic stellate cells (PSCs), cancer-associated fibroblasts (CAFs), myeloid cells, regulatory T cells, B cells, endothelial cells, and neuronal cells. Together, these cells create a TME marked by fibrosis, hypoxia, and immune suppression (15). As research progresses, it becomes clearer that the TME is critical in tumor initiation and response to treatment. Targeting TME components offers promising avenues for clinical therapies.

1) Tumor Cells.

Tumor cells are a key element of the TME. They induce substantial molecular, cellular, and physical alterations in surrounding tissues, facilitating tumor growth and progression (16). Their uncontrolled proliferation and resistance to apoptosis contribute to tumor formation.

2) Tumor cells can secrete exosomes rich in circRNA, influencing immune evasion mechanisms. For instance, circRNA-002178 secreted by hepatocellular carcinoma (HCC) upregulates PD-L1 expression through the miR-34 axis, promoting CD8+ T cell exhaustion and enabling tumor cells to evade immune surveillance. Additionally, certain circRNAs can interact with the extracellular matrix (ECM), facilitating tumor cell migration and invasion, which further exacerbates immune suppression in the TME.

3) CAFs:

Cancer-associated fibroblasts (CAFs) actively promote tumor growth (17, 18). CAFs are a phenotype induced and activated by signals from cancer cells (19). CAFs react to tissue damage inflicted by cancer cells and produce various components that support tumor growth. They also play a vital role in ECM remodeling, immune reprogramming, and TME metabolism (20). Specific subtypes of stromal and immune cells are identified as critical for creating a pro-tumoral microenvironment in metastatic lesions, with RGS5+ CAFs facilitating an environment favorable for cancer cell growth and metastasis (21).

4) CAFs are a type of fibroblast induced and activated by cancer cells that influence tumor immune responses by secreting matrix components and remodeling the ECM. CircRNAs secreted by CAFs can modulate ECM structure and affect immune cell infiltration, for example, by enhancing T cell suppression through the expression of specific cytokines. This regulatory mechanism is particularly significant in certain solid tumors, such as pancreatic cancer, where CAFs play a key role in fibrosis and immune evasion.

5) Immune Cells:

Immune cells, such as T cells, B cells, natural killer (NK) cells, and phagocytic cells, are critical components of the TME. Their main role is to monitor and eliminate abnormal cells, including tumor cells. The TME utilizes various mechanisms to suppress immune cell activity, such as expressing inhibitory receptors like CTLA-4, PD-1, and PD-L1, which impair normal immune function. Moreover, new immune checkpoint inhibitors (ICBs) can target LAG-3 to restore some normal immune functions (22). Inhibitory cytokines such as TGF-β and VEGFA can further affect immune cells, facilitating immune escape (23).

6) Within the TME, immune cells such as T cells, B cells, NK cells, and phagocytes are responsible for monitoring and eliminating abnormal cells under normal conditions. However, circRNAs can regulate the activity of these immune cells via exosomes, diminishing their anti-tumor effects. For example, circUHRF1 secreted by hepatocellular carcinoma upregulates TIM-3 expression via the miR-449c-5p axis, inhibiting NK cell function and promoting tumor immune evasion. Other circRNAs have also been found to weaken T cell activity and reduce the efficacy of anti-tumor immune responses by regulating T cell exhaustion pathways.

7) Vascular System:

The vascular system in the TME is essential for nutrient supply and the infiltration of immune cells. However, tumors can promote abnormal blood vessel formation, resulting in inadequate blood flow and oxygen supply, which restricts immune cell entry and reduces their activity. This creates a relatively isolated and protected environment for tumors, shielding them from immune attacks. Studies indicate that T cells can migrate and extravasate in response to gradients of rhCXCL11 and rhCXCL12 through HUVEC-derived vascular tubes cultured under specific conditions, including collagen barriers and chemotactic factors (24). The vascular system is crucial for supporting these experimental observations.

8) The vascular system is critical in the TME for supplying necessary nutrients and oxygen to tumor cells. However, tumor cells can induce abnormal angiogenesis, leading to insufficient blood supply and hypoxic conditions that limit effective immune cell infiltration. CircRNAs help maintain an immune-secluded environment for tumor cells by regulating angiogenesis-related signals within the TME. For example, certain circRNAs can indirectly inhibit T cell and NK cell infiltration by upregulating the expression of angiogenesis-related factors, thereby creating favorable conditions for tumor growth.

## Immunosuppression and escape mechanism of TME

Within the complex interactions of the TME, there exist mechanisms of immune suppression and immune evasion, allowing tumors to evade immune system control. These mechanisms include the expression of immune inhibitory receptors such as CTLA-4, PD-1, and PD-L1 to suppress immune cell function (22). Currently, the next generation of DC-derived exosomes (DEX) -engineered vaccines is under development with the aim of enhancing the expression of stimulatory molecules on the surface of DEXs while reducing the expression of immune-regulatory molecules such as PD-L1 and Regulatory T cells (Tregs) to mitigate immune suppression (25). Notably, Treg cells specifically express and secrete certain crucial circRNAs, such as circFoxp3 and circHelz. These circRNAs, when transferred into target cells, can inhibit cell proliferation and promote apoptosis. For instance, in oral squamous cell carcinoma (OSCC), the transfer of has_circ_0069313 from exosomes into Treg cells inhibits Foxp3 degradation induced by miR-325-3p, thereby promoting immune evasion (26, 27). Furthermore, immunosuppressive cytokines like TGF-β and IL-10 play a role in this mechanism (28). In immune cell exclusion, the ECM and chemical signals within the TME can impede immune cell infiltration, leading to immune suppression. For example, in the absence of CD200R, tumor-associated myeloid cells (TAMCs) increase the expression of C-C motif chemokine ligand 24 (CCL24), resulting in enhanced eosinophil infiltration and thereby boosting anti-tumor capabilities (29).

## Exosomes and circRNAs

### Origin, structure and function of exosomes

**Figure 1:**
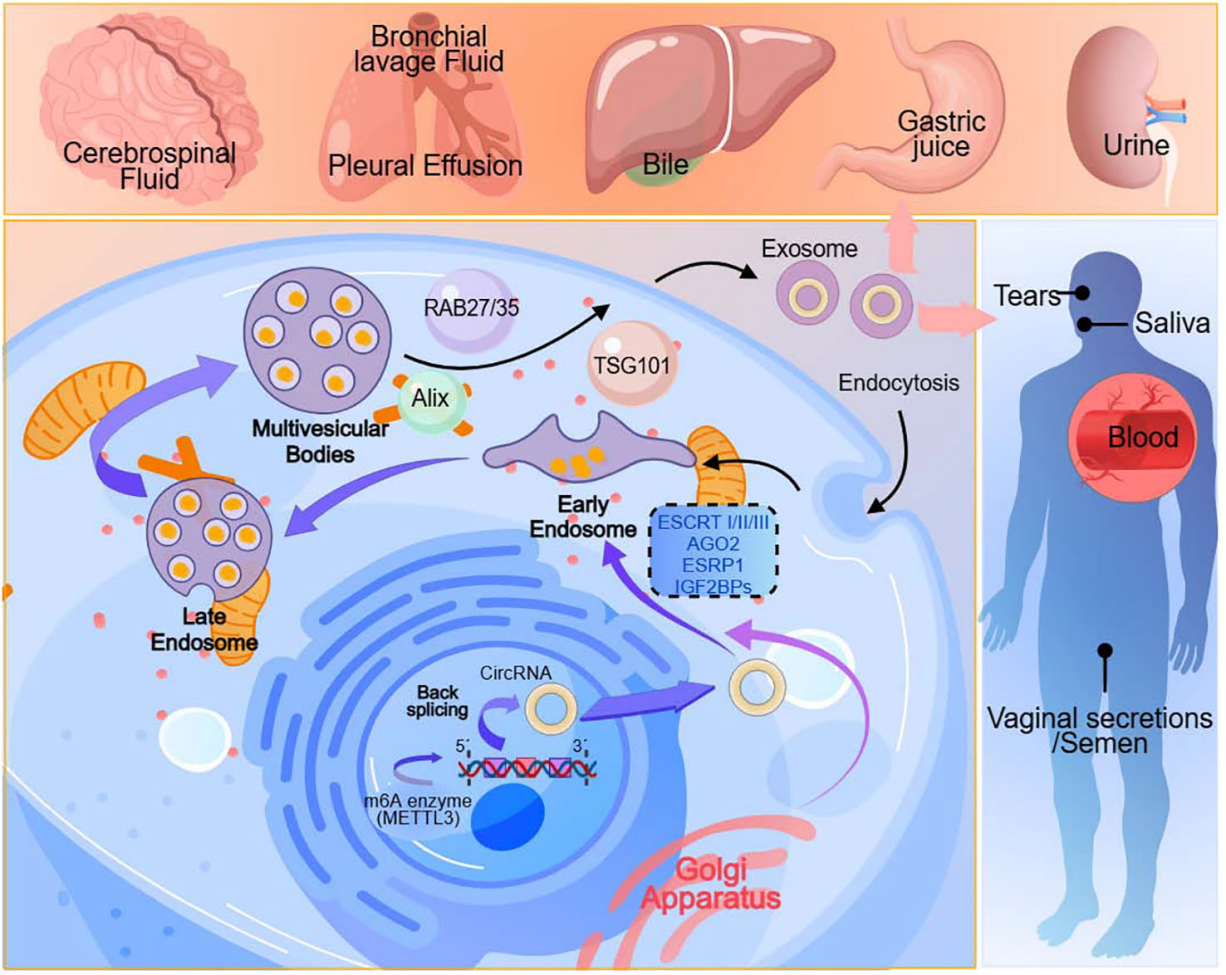
Exosomes originate from cells. They are present in various body fluids, including urine, saliva, blood, bile, tears, pleural effusion, bronchial lavage fluid, cerebrospinal fluid, vaginal secretions, semen, gastric juice and etc.

The biogenesis of exosomes is an exceedingly complex process. The smallest constituents of these cell-derived vesicles are known as exosomes, and they can be found in various bodily fluids, including urine, saliva, blood, bile, tears, pleural effusion, bronchial lavage fluid, cerebrospinal fluid, vaginal secretions, semen, gastric juice and etc (30)(**Figure 1**). Exosomes, nano-sized vesicles, contain DNAs, mRNAs, microRNAs, circRNAs, metabolic products, lipids, cytoplasm, and cell surface proteins, enabling intercellular communication and exhibiting therapeutic potential akin to that of stem cells (31). It indicates that exosomes originate from intracellular vesicles formed through endocytosis of the cell membrane. They undergo various maturation stages, such as intraluminal vesicles and multivesicular bodies, followed by directed assembly, intracellular migration, and ultimately, fusion with the cell membrane, releasing their contents into the extracellular interstitium via exocytosis. The contents of exosomes can reflect the cell’s source and provide insights into the cellular status and tissue changes under pathological conditions (32). Exosomes play a crucial role in various aspects of vascular regeneration, tissue repair, and inflammation regulation during the wound healing process in diabetes (33). Furthermore, their intrinsic biocompatibility, stability, drug-loading capacity, and minimal immunogenicity position exosomes as competitors in the drug delivery system (DDSs) for osteoarthritis (OA) treatment (34). It suggests that exosome-based therapies can enhance wound healing by increasing vascularization, re-epithelialization, collagen deposition, and reducing scar formation, providing a promising direction for future clinical treatments (35).

### Biological properties and functions of circRNAs

CircRNAs, a type of non-coding RNA, are characterized by its closed circular structure, lacking the 5’ or 3’ ends and the PolyA tail (36–38). Due to theirs stability and resistance to RNA exonucleases or RNase R degradation, circRNAs hold significant potential as a biomarker with applications in cancer diagnosis, treatment, and prognosis (39, 40). Additionally, circRNAs can influence cancer through the regulation of glucose metabolism. While most circRNAs promote carcinogenesis by modulating cancer glucose metabolism (41), a minority of circRNAs inhibit the onset and progression of cancer through glucose metabolism regulation (42). CircRNAs exhibit high expression abundance, often displaying tissue-specific and developmental-stage-specific expression patterns. It has identified certain circRNAs as key metabolic regulators. When combined with previous studies suggesting circRNAs as a potential biomarker for polycystic ovary syndrome (PCOS), this discovery could offer attractive therapeutic targets for PCOS metabolic consequences (43). CircRNAs can influence gene expression and cancer metastasis by interacting with miRNAs. Additionally, circRNAs can act as competitive endogenous RNAs (ceRNAs) to inhibit miRNA-mediated suppression of specific mRNAs, thus inducing or suppressing oral cancer metastasis (44).

### The packaging of circRNAs into exosomes

There is increasing evidence to suggest that circRNAs are abundantly present within exosomes. Tumor cells are capable of generating and releasing a large quantity of exosomes, far surpassing normal cells. Tumor-derived circRNAs are primarily packaged into exosomes and subsequently disseminated into the circulatory system (45). This results in the formation of “exosomal circRNA,” characterized by its high stability and abundant content, making it an increasingly significant biomarker and therapeutic target in cancer (46).

**Figure 2:**
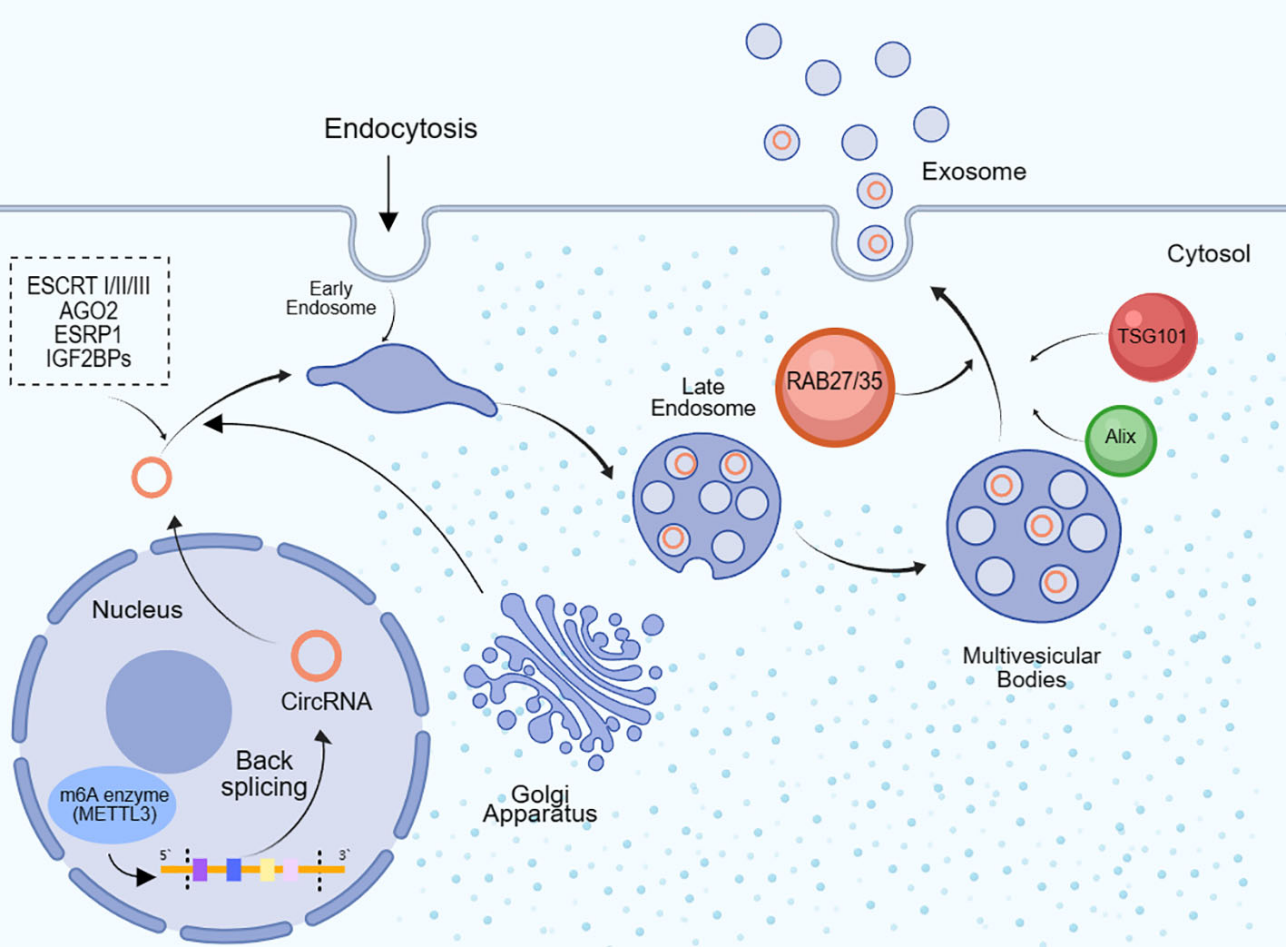
CircRNAs are packaged into exosomes. After circRNAs are formed through the backward splicing of 5’ and 3’ splice sites in the cell nucleus, they are transported to the cytoplasm and then go through early endosomes, late endosomes and multivesicular bodies, before being packaged into exosomes and secreted extracellularly. Once entering the cytoplasm, circRNAs can bind to RNA binding proteins (RBPs) like AGO2 and ESRP1, and then be internalized into endosomes mediated by the ESCRT complex. Additionally, the m6A methyltransferase METTL3 can facilitate circRNA circularization and recruit RBPs like IGF2BPs to stabilize circRNAs. The Golgi apparatus is also involved in the early transportation of circRNAs from the nucleus to the cytoplasm. RAB family GTPases including RAB27, RAB35, TSG101 and Alix can affect circRNA packaging by modulating exosome biogenesis and secretion.

The mechanisms underlying the packaging of circRNAs into exosomes are diverse, and we provide a concise overview here(**Figure 2**). CircRNAs can be directly packaged into exosomes by binding with RNA-binding proteins (RBPs), such as AGO2 and ESRP1 (47). Cells producing circRNAs selectively take up cytoplasmic circRNAs through endocytosis and then expel them into exosomes through exocytosis (48). The Endosomal Sorting Complex Required for Transport (ESCRT) complex can mediate the internalization of small vesicles and the formation of multivesicular bodies, thereby regulating the entry of circRNAs into exosomes (49). ESCRT-I, ESCRT-II, and ESCRT-III complexes collectively promote this process. Cells can also engulf circRNAs through phagocytosis, and these engulfed circRNAs can subsequently be released into exosomes (50). Additionally, certain proteins like RAB35, TSG101, Alix, among others, can influence circRNAs packaging by modulating exosome biogenesis and release. RAB family GTPases, such as RAB27 and RAB35, can promote exosome generation and release, thereby mediating circRNAs packaging (51). CircRNAs can also be released into the extracellular matrix following tumor cell necrosis or lysis, subsequently being absorbed and packaged into exosomes (52). Some studies have also found that m6A modification plays a role in circRNAs exosomal sorting, as m6A modification can facilitate circRNAs production. The m6A enzyme METTL3 can promote circRNAs circularization and stabilize circRNAs by recruiting RNA-binding proteins IGF2BPs (53). For instance, exosomes derived from M1 macrophages inhibit the expression of human methyltransferase-like 14 (METTL14) in hepatocellular carcinoma (HCC) cells by transferring miR-628-5p, while METTL14 promotes m6A modification of circFUT8, facilitating its cytoplasmic export (54). Additionally, enzymatic cleavage, changes in methylation levels, recombination of cytoskeletal proteins such as actin, and cellular stress conditions such as radiation and hypoxia can also affect the packaging of circRNAs into exosomes.

### The role of exosomes and their contents in the tumor microenvironment

Exosomes have emerged as a widely studied field of interest in recent years. They are small vesicles containing various biological molecules, including RNA, proteins, and lipids. Exosomes play diverse roles within the tumor microenvironment (TME), encompassing information transmission, immune regulation, and modulation of tumor cell behavior (55–57). It suggests that circRNAs within exosomes may have a significant role in tumor immune regulation. These circRNAs can be transported via exosomes to modulate the coordinated functions within the TME, influencing immune cell functionality and immune responses, thus impacting tumor growth and progression (58) (**Table 1**). For instance, circRNAs within exosomes can enhance the immunosuppressive activity of tumor-associated myeloid-derived suppressor cells (MDSCs). Studies indicate that exosomes from MDSCs contain circMID1, which upregulates MID1 expression by adsorbing miR-506-3p, thereby enhancing the immunosuppressive activity of cytokines secreted by MDSCs (59).

**Table 1:** The effect of circRNAs in exosomes on tumor development and targeting miRNAs and signaling pathways in tumor tissue.

CircRNA	Cancer Type	Target miRNA	Signaling Pathway	Function	Reference
cSERPINE2	Breast cancer	miR-513a-5p	miR-513a-5p/MALT1/NF-κB pathway	Enhance IL-6 secretion, promote breast cancer proliferation and invasion	(20)
circMID1	-	miR-506-3p	-	Enhance immunosuppressive cytokine secretion by MDSCs	(66)
circ_0008285	-	miR-4644	miR-4644/LDLR axis	Regulate lipid metabolism	(68)
circ_MMP2	Hepatocellular carcinoma	miR-136-5p	miR-136-5p/MMP2 axis	Promote HCC metastasis	(69)
circ_102481	Non-small cell lung cancer	miR-30a-5p	circ_102481/miR-30a-5p/ROR1 axis	Enhance tumor invasion and metastasis	(71)
hsa_circ_000200	Gastric cancer	miR-4659a/b-3p	miR-4659a/b-3p/HBEGF/TGF-β/Smad axis	Enhance gastric cancer invasion and metastasis	(72)
circFARSA	Non-small cell lung cancer	-	PTEN/PI3K/AKT pathway	Induce M2 polarization of TAMs, promote lung cancer metastasis	(74)
circSAFB2	Renal cell carcinoma	miR-620	miR-620/JAK1/STAT3 axis	Mediate M2 TAM polarization, promote renal cancer metastasis	(75)
circ0048117	Esophageal squamous cell carcinoma	miR-140	-	Cause TAM polarization into M2 type, promote cancer progression	(76)
hsa_circ_0001610	Endometrial cancer	miR-139-5p	-	Upregulate cyclin B1 expression, reduce radiosensitivity	(77)
circWDR25	Hepatocellular carcinoma	miR-4474-3p	miR-4474-3p/ALOX15 axis	Induce EMT, promote HCC metastasis	(79)
circCCAR1	Hepatocellular carcinoma	-	-	Induce CD8+ T cell dysfunction	(81)
circTRPS1	Bladder cancer	miR141-3p	circTRPS1/miR141-3p/GLS1 axis	Induce CD8+ T cell exhaustion	(82)
circZNF451	Lung adenocarcinoma	-	-	Induce CD8+ T cell exhaustion, affect cancer progression	(83)
circTMTC3, circFAM117B	-	miR-142-5p	miR-142-5p/PD-L1 axis	Increase PD-L1 expression, reduce T cell activity, lead to immune evasion	(84)
circRNA-002178	Lung adenocarcinoma	miR-34	-	Enhance PD-L1 expression, induce T cell exhaustion, promote cancer progression	(85)
circ_0073453	Gastric cancer	miR-146a-5p	-	Enhance PD-L1 expression, reduce CD8+ T cell cytotoxicity	(86)
circUBAP2	Pancreatic adenocarcinoma	-	-	Promote immune evasion	(87)
circUHRF1	Hepatocellular carcinoma	miR-449c-5p	miR-449c-5p/TIM-3 pathway	Inhibit NK cell function, lead to immune suppression	(88)
circGSE1	Hepatocellular carcinoma	-	-	Promote HCC immune evasion	(87)

CircRNA	Cancer Type	Target Molecule	Involved Signaling Pathways	Function Description	
circFARSA	Non-small Cell Lung Cancer	miR-620	PTEN/PI3K/AKT Pathway	Induces TAMs polarization to M2, promoting lung cancer metastasis.	
circSAFB2	Renal Cell Carcinoma	miR-513a-5p	JAK1/STAT3 Pathway	Regulates TAMs polarization, promoting renal cancer metastasis.	
circ0048117	Esophageal Squamous Cell Carcinoma	miR-140	Interacts with IL-6	Promotes TAM polarization to M2, aiding cancer progression.	
circZNF451	Lung Adenocarcinoma	miR-338	TRIM56/FXR1 Complex Pathway	Induces CD8+ T cell exhaustion, impacting cancer progression.	

## The dual regulatory role of exosomal circRNAs in tumor metastasis

Exosomal circRNAs regulate the interaction between immune cells and tumor cells as well as influence the infiltrative and invasive abilities of tumor cells (44). Cross-talk between tumor cells and immune cells requires circRNAs as signal molecules to mediate (60). These circRNAs can regulate immune cell responses through various pathways, among which the signaling pathways associated with tumor metastasis are particularly important. They can promote tumor metastasis, but can also inhibit tumor metastasis. Studies have found that high levels of circ_MMP2 and low levels of miR-136-5p are associated with lower overall survival rates in HCC patients, which can facilitate tumor metastasis (61). However, some circRNAs may also inhibit tumor metastasis. They slow down the metastatic process of tumors by inhibiting immune escape pathways or reducing the invasiveness of tumor cells. For instance, circNEIL3 exerts its metastasis inhibitory effect through direct interaction with the oncoprotein Y-box binding protein 1 (YBX1), thereby promoting Nedd4L-mediated YBX1 proteasomal degradation to retard tumor metastasis (62). Hence, the role of exosomal circRNAs in tumor metastasis is intricate and diverse. In the process of tumor metastasis, infiltration and invasion of tumor cells are crucial steps. Exosomal circRNAs play a vital part during this process by modulating tumor cell behavior, thereby influencing their invasive and infiltrative abilities. Exosomal circRNAs can impact a tumor’s invasive capacity by upregulating invasion-related genes. Studies have uncovered that lung cancer cell-secreted exosomes contain circ_102481 which can enhance tumor cell invasion and migration by sponging miR-30a-5p to upregulate ROR1 expression (63). Hsa_circ_000200 can increase the expression of HBEGF and TGF-β/Smad by absorbing miR-4659a/b-3p, thus enhancing gastric cancer cell proliferation, migration and invasion (64).

CircRNAs can also enable tumor cells to obtain invasive abilities by regulating epithelial-mesenchymal transition (EMT). Some circRNAs may facilitate tumor cell EMT by modulating the expression of EMT-related genes, thereby strengthening their infiltration capacity. For example, both exogenous and hematopoietic stem cell-derived exosomal circWDR25 can induce HCC cell EMT through the sponge-like miR-4474-3p to regulate ALOX15 expression, ultimately promoting HCC cell proliferation and invasion (65).

Future research needs to delve deeper into the specific mechanisms of different circRNAs and their relative importance in tumor metastasis. This will facilitate the development of novel therapeutic strategies to intervene in metastatic processes for improved survival of cancer patients.

## Regulation of immune responses by exosomal circRNAs

### Exosomal circRNAs in mediating tumor-associated macrophage activity and function

Exosomal circRNAs can modulate the polarization of tumor-associated macrophages (TAMs) within the tumor microenvironment, either promoting or inhibiting tumor development. It has revealed that circFARSA derived from non-small cell lung cancer cells can induce M2 polarization of TAMs by ubiquitinating and degrading PTEN, further activating the PI3K/AKT signaling pathway, thereby promoting NSCLC metastasis (66). Exosomal circSAFB2 mediates M2 polarization of TAMs through the miR-620/JAK1/STAT3 axis, promoting renal cell carcinoma metastasis (67). Hypoxic tumor cells can secrete exosomes containing circ0048117, which, by adsorbing miR-140, induces TAM polarization into an M2 anti-inflammatory phenotype (68). Additionally, certain circRNAs can regulate the phagocytic activity of TAMs, including circPHF14 and circAGO2. Some circRNAs can also impact the ability of TAMs to release pro-tumor factors. Studies have found that hsa_circ_0001610 is abundant in the exosomes of M2-polarized macrophages. Hsa_circ_0001610 acts as a competitive endogenous RNA for miR-139-5p, upregulating the expression of cell cycle protein B1, a crucial factor in driving radioresistance in various cancers by regulating the cell cycle (69). Mechanistically, circRNAs primarily modulate TAM-related signaling pathways through the miRNA sponge effect. For example, circRNA cSERPINE2, derived from the tumor gene SERPINE2, shuttles into tumor-associated macrophages. By interacting with miR-513a-5p, cSERPINE2 significantly increases MALT1 levels, enhancing the secretion of interleukin-6 (IL-6). IL-6, by increasing JAK2-STAT3 pathway activation, promotes the proliferation and invasion of breast cancer cells (70). Furthermore, many studies indicate that targeting circRNAs associated with TAMs can improve the tumor microenvironment, enhancing the effectiveness of tumor immunotherapy.

### Exosomal circRNAs and T cells

Tumor cells can secrete exosomes containing circRNAs, which, when taken up by immune cells, can influence the immune response. T cells are key players in the human immune response (71, 72). Certain tumor-derived exosomal circRNAs can downregulate T cell proliferation and activation, inhibiting the maturation of immune-activating cells such as dendritic cells (73). For instance, HCC cells secrete circCCAR1 in a heterogeneous nuclear ribonucleoprotein A2/B1-dependent manner. Exosomal circCCAR1 is absorbed by CD8+ T cells, leading to CD8+ T cell dysfunction through the stabilization of PD-1 protein and the consequent inhibition of dendritic cell maturation (74). Exosomal circTRPS1 from BCa cells regulates intracellular reactive oxygen species (ROS) balance and CD8+ T cell exhaustion via the circTRPS1/miR141-3p/GLS1 axis, while also inhibiting dendritic cell maturation (75). Moreover, exosomal circRNAs can directly act on T cells, inhibiting their activity, promoting apoptosis and exhaustion. They may also suppress T cell activity by regulating immune escape signaling pathways such as PD-1/PD-L1 and CTLA-4, leading to T cell dysfunction and tumor cell escape. For instance, in lung adenocarcinoma, exosomal circZNF451 induces the exhaustion of cytotoxic CD8+ T cells, affecting cancer progression (76). Overexpression of circTMTC3 and circFAM117B can increase PD-L1 expression by adsorbing miR-142-5p/PD-L1 axis, thereby reducing T cell activity and causing T cell dysfunction, resulting in immune escape (77). CircRNA-002178 in lung adenocarcinoma can be delivered to CD8+ T cells via exosomes, enhancing PD-L1 expression in cancer cells through miR-34 sponge effect, leading to T cell exhaustion and promoting cancer development (78). Exosomal circRNAs can influence T cells in various ways in different cells. Circ_0073453 can regulate IL-8 secretion from gastric cancer stromal stem cells, increasing gastric cancer cell PD-L1 expression and reducing the cytotoxicity of CD8+ T cells (79). Studies have found that the overexpression of CXCR4 and ZEB1 genes in pancreatic adenocarcinoma (PAAD) tissues is regulated by circUBAP2. The expression of these two genes is associated with Tregs and exhausted T cell levels, as well as the expression of CTLA-4 and PD-1. CXCR4 and ZEB1 proteins promote T cell exhaustion, thereby facilitating immune escape (80).

### Exosomal circRNAs and NK cells

**Figure 3:**
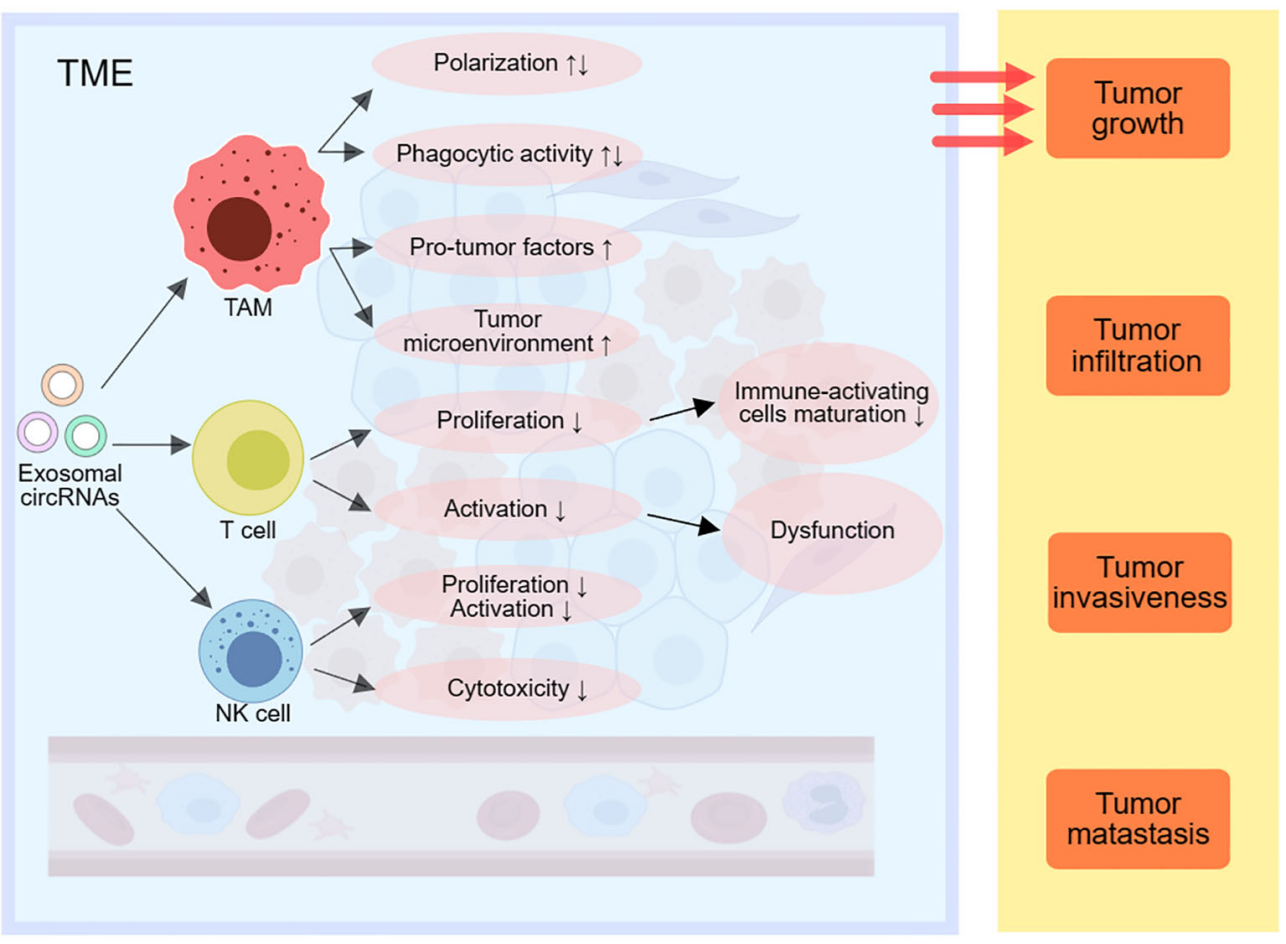
Exosomal circRNAs can modulate immune responses by affecting TAMs, T cells and NK cells in the TME, thereby promoting tumor growth, infiltration, invasion and metastasis. TME, tumor-associated macrophage; NK cells, natural killing cells; TME, tumor microenvironment; EMT, epithelial-mesenchymal transition.

NK cells are also essential cells in the human immune response (71, 72). Certain tumor-derived exosomal circRNAs can downregulate NK cell proliferation and activation (73). It has found that circUHRF1 derived from HCC cells’ exosomes can be taken up by NK cells, inhibiting NK cell activity by upregulating TIM-3 expression through miR-449c-5p degradation, leading to NK cell dysfunction and immune suppression (81). Exosomal ciRS-133 can suppress NK cell cytotoxicity by downregulating miR-133 levels. Meanwhile, exosomal circPDE8A, through competitive binding with miR-338, upregulates MACC1 and MET expression, reducing natural killer cell cytotoxicity, and promoting PDAC infiltration and growth (**Figure 3**).

### Clinical applications of exosomal circRNAs

**Figure 4:**
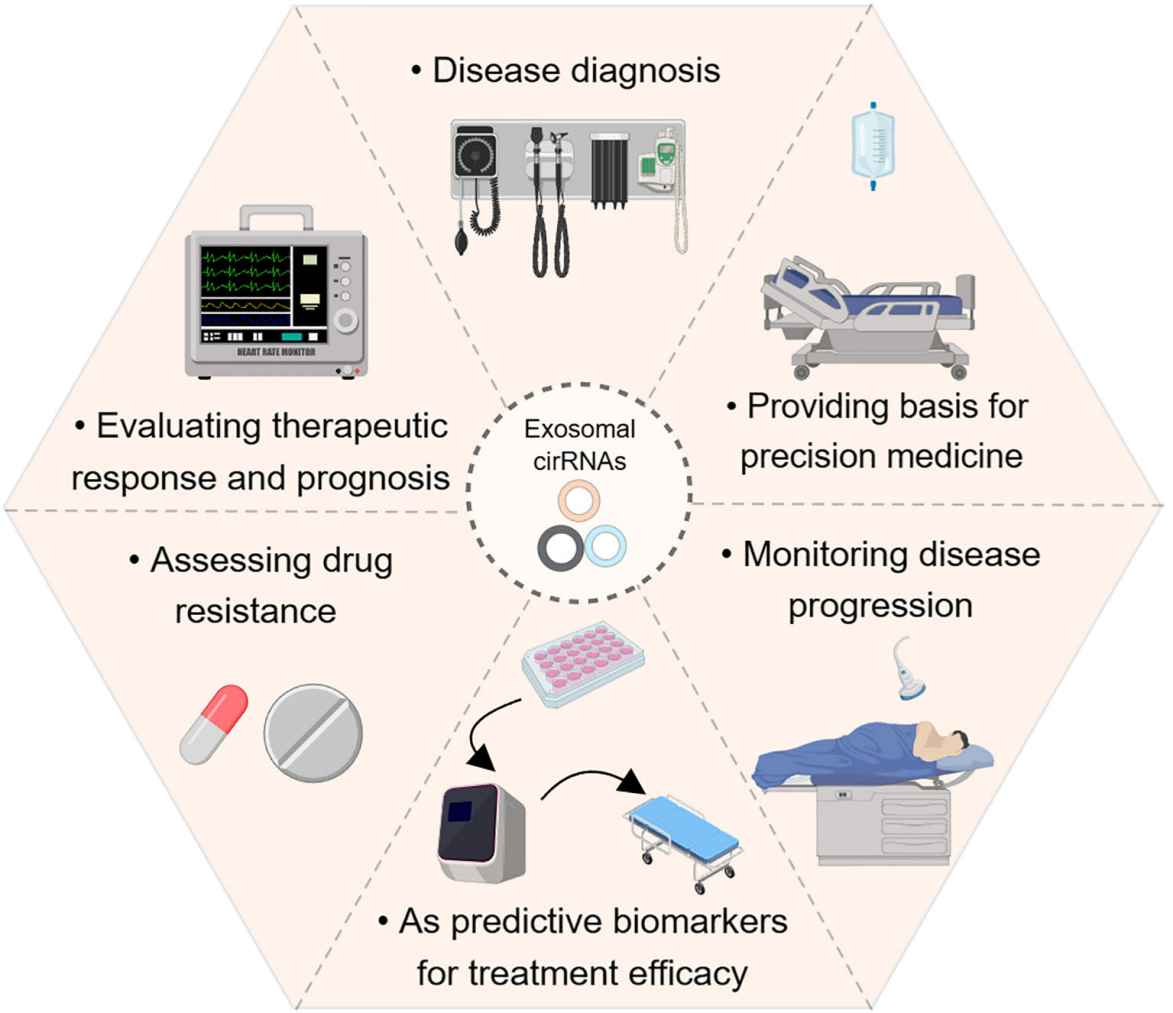
The clinical application of circRNAs in exosomes and their impact on tumor cell function.

Research indicates that exosome-carried circular RNAs (circRNAs) play a significant role in immune regulation within TME. Due to their highly stable structure and specific expression patterns, circRNAs hold potential as non-invasive tumor biomarkers, suitable for precise tumor screening and diagnosis, and are increasingly showing clinical value in immunotherapy (11) (**Figure 4**). Exosomes, serving as intercellular transport vehicles, can deliver circRNAs to tumor cells and immune cells, altering their biological functions and regulating intercellular signaling within the TME. Through this mechanism, circRNAs in exosomes affect tumor-related signaling pathways, modulating the body’s anti-tumor immune response within the TME (82). Specifically, many circRNAs act as competing endogenous RNAs (ceRNAs) that bind to specific microRNAs (miRNAs), blocking miRNA access to their target genes. This indirectly upregulates the expression of relevant genes, influencing signaling pathways related to cancer progression, cell proliferation, and apoptosis. For instance, studies have found that exosome-enriched circNEIL3 can be secreted by glioblastoma cells into tumor-associated macrophages (TAMs), stabilizing the expression of IGF2BP3 and upregulating pro-inflammatory factors like IL-10 and ARG1, inducing M2 immune suppressive polarization, and ultimately leading to tumor immune evasion (83). Furthermore, circRNAs also show promise in immune checkpoint blockade (ICB) therapy. For example, circUHRF1 can induce resistance to anti-PD-1 therapy, reducing its efficacy. This suggests that targeting specific circRNAs may improve the tumor microenvironment and enhance the immune sensitivity of tumor cells, thereby strengthening the body’s anti-tumor immune response (84). For instance, circUHRF1 found in exosomes can induce resistance to anti-PD-1 therapy, reducing its efficacy (81). Targeting key circRNAs can improve the tumor microenvironment, enhance tumor cell immune sensitivity, and thereby boost the body’s anti-tumor immune response (85). For example, circGSE1 found in exosomes can induce the proliferation of regulatory T cells (Tregs), promoting immune evasion in hepatocellular carcinoma (86). Additionally, targeting specific circRNAs could improve the tumor microenvironment, enhance the efficacy of chemotherapy and radiotherapy, and reduce side effects, thereby optimizing treatment strategies (85). CircRNAs such as circPRMT5, circPIP5K1A, and circHIPK3 have been shown to promote proliferation, metastasis, and drug resistance in colon cancer cells. Consequently, they are not only stable tumor biomarkers but also beneficial for precise tumor screening and prognostic assessment due to their ease of detection (87). Monitoring dynamic changes in the levels of key circRNAs can enable real-time assessment of the tumor immune microenvironment and prognostic risks (88).

However, despite the promising potential of circRNAs in cancer treatment, several challenges remain in their practical application. First, the expression patterns of circRNAs vary significantly across different cancer types and stages, limiting their universality across various cancers. Additionally, while circRNAs exhibit high stability in bodily fluids, the mechanisms underlying their loading into exosomes are not fully understood, affecting their targeted application in the tumor microenvironment. Future research should further investigate the specific expression patterns of circRNAs in various cancers and their regulatory mechanisms within the TME. Moreover, large-scale clinical trials are needed to validate the effectiveness of circRNAs as diagnostic biomarkers and therapeutic targets. Addressing these challenges will facilitate the transition of circRNAs from the laboratory to clinical application, positioning them as an innovative strategy in cancer treatment.

## Conclusion and prospect

Research indicates that circRNAs within exosomes play multifaceted roles in immune regulation within the TME. Exosomes can deliver circRNAs to tumor cells or immune cells, thereby influencing their biological functions. circRNAs affect immune cell activity through various mechanisms, regulate immune evasion pathways, alter tumor cell phenotypes, and modulate inflammatory responses. As signaling molecules, the transfer of circRNAs via exosomes has become a key mediator of local tumor immunity within the TME. These findings not only highlight the diversity of circRNAs in tumor immune processes but also provide new insights into tumor immune evasion and progression. Exosomal circRNAs hold significant potential as targets for immunotherapy, offering promising clinical applications. By regulating key circRNAs within exosomes, we can modulate immune cell responses and enhance the body’s immune defense against tumors. This approach introduces new strategies for cancer immunotherapy, particularly when combined with ICBs or CAR-T cell therapies, potentially resulting in synergistic effects. Additionally, exploring exosomal circRNAs as precise diagnostic and therapeutic biomarkers will advance personalized cancer treatment.

However, current research on circRNAs in cancer immunotherapy faces several potential limitations that need to be addressed in future studies. Firstly, the expression patterns of circRNAs vary significantly across different cancer types and stages, limiting their universal applicability in various cancers. Therefore, there is an urgent need to systematically identify and validate key circRNAs in a broader range of cancer types. Additionally, although circRNAs exhibit high stability in bodily fluids, the mechanisms by which they are loaded into exosomes remain unclear, posing challenges for their targeted application within the TME. On a technical level, existing methods for detecting and quantifying exosomal circRNAs are not standardized, necessitating the development of more sensitive and specific detection techniques to enhance the reliability and reproducibility of circRNA clinical applications.

Another significant knowledge gap is that many studies on circRNA functions are primarily conducted *in vitro*, with insufficient *in vivo* validation. Therefore, future research should focus more on elucidating the mechanisms of circRNAs in animal models to confirm their functions under actual physiological and pathological conditions. Moreover, the specific interactions between circRNAs and immune regulatory signaling pathways are not yet fully understood, necessitating in-depth molecular biology studies to uncover these mechanisms.

To address these knowledge gaps, future research should focus on several key areas. Firstly, developing and optimizing high-throughput sequencing technologies, quantitative PCR, *in situ* hybridization, and other detection methods is essential for accurately identifying and quantifying circRNAs within exosomes, thereby establishing standardized detection protocols and procedures. Secondly, expanding the scope of circRNA research to include a wider variety of cancer types is crucial. This involves systematically identifying and characterizing key circRNAs and their functions across different cancers to evaluate their potential as universal biomarkers and therapeutic targets. Additionally, enhancing functional studies of circRNAs in animal models is necessary to validate their roles in regulating immune responses and tumor progression, ensuring that *in vitro* findings are applicable *in vivo*. Concurrently, in-depth investigations into the interactions between circRNAs and immune regulatory signaling pathways (such as NF-κB, STAT, PI3K/AKT) are needed to elucidate their specific molecular mechanisms, providing a theoretical foundation for targeted regulation.

Additionally, conducting large-scale clinical trials to validate the efficacy and safety of circRNAs as diagnostic tools, biomarkers, and therapeutic targets is crucial for their clinical application. Exploring the combined use of circRNA regulation strategies with existing immunotherapies, such as ICBs and CAR-T cell therapies, will help assess their synergistic effects and optimize personalized treatment plans. Finally, developing efficient circRNA-targeted delivery systems is essential to ensure their stability and specificity within the body, thereby enhancing therapeutic outcomes and minimizing potential side effects. By thoroughly exploring these research directions, we can address current research limitations, advance the clinical application of circRNAs in cancer immunotherapy, and promote the development of precision medicine. Overcoming these challenges will not only enhance the potential of circRNAs as immune regulatory molecules but also provide cancer patients with more effective and personalized treatment options.
